# Metabolic syndrome in children and teenagers: worth assessing it, but how?

**DOI:** 10.1590/2359-3997000000249

**Published:** 2017-01-01

**Authors:** Gilberto J. Paz-Filho

**Affiliations:** 1 John Curtin School of Medical Research The Australian National University Canberra ACT Australia John Curtin School of Medical Research, The Australian National University, Canberra, ACT, Australia

Reaven has defined metabolic syndrome (at that time, “syndrome X”) as a clustering of risk factors for coronary heart disease and type 2 diabetes that include elevated glucose, hypertension, central obesity, and dyslipidemia (1). Since then, significant breakthroughs have been achieved in the field, but there is still much controversy regarding its diagnosis, and whether it should be considered as a single disease at all. Several organizations have proposed different diagnostic criteria, which have in common many caveats, such as the fact that they treat each component in a dichotomous way (above or below a cutoff) rather than in a continuous manner. Furthermore, equal weight toward the diagnosis of the metabolic syndrome is given to each criterion. After a couple of decades, Reaven himself questioned the clinical utility of metabolic syndrome as a diagnostic category (2), and some suggested that the time of metabolic syndrome as a condition has already passed (3). Nevertheless, much research on the diagnosis of metabolic syndrome is being published: up to January 23, 2017, 24,173 abstracts could be found on PubMed, when searching for the MeSH term “metabolic syndrome X”.

Since children are also affected by all components of metabolic syndrome, it is nothing but natural to expand current research to a younger population. In this issue of AE&M, two interesting cross-sectional studies propose to determine different tests to diagnose metabolic syndrome in children and teenagers. In particular, Madeira and cols. aim to determine a cutoff value for serum leptin levels in prepubertal children, as a predictor of metabolic syndrome (4). Conversely, Stroescu and cols. provide a carotid intima media thickness (CIMT) cutoff value in children and teenagers, which intends to predict an increased risk of metabolic syndrome (5).

In the paper by Madeira and cols., cross-sectional data from 340 Brazilian prepubertal children between ages 5-11 years were analyzed. As widely replicated in previous studies, several biomarkers were altered in the obese/overweight groups, indicating increased risk for cardiovascular disease and type 2 diabetes (6). In other words, children in those groups were more likely to have metabolic syndrome. In fact, the prevalence of metabolic syndrome (adapted IDF criteria) was higher in the group of obese children (n = 33, 20%), when compared to what was observed in overweight (n = 1, 2%) and in age-matched, normal weight children (0%). However, the most significant finding of that study is the determination of a serum leptin cutoff value of 13.4 ng/mL that indicates the presence or absence of metabolic syndrome.

Leptin has been proposed as a biomarker of metabolic syndrome due to its effects that contribute to the development of type 2 diabetes mellitus and cardiovascular disease. It has atherogenic, thrombotic, angiogenic, and proinflammatory effects. Furthermore, it increases oxidative stress, and promotes vascular smooth muscle hypertrophy (7,8). Therefore, leptin is associated with the components of metabolic syndrome, and can contribute to the development of cardiovascular disease and diabetes. However, the direct role of leptin in the pathogenesis of some components of metabolic syndrome is controversial. Although leptin participates in the activation of the sympathetic system, it is unclear whether it is a causal agent of hypertension in humans (9). In case of plasma glucose levels (and insulin sensitivity), leptin seems to be beneficial only in the absence of leptin resistance (10,11), which could deter its use as a widespread biomarker of metabolic syndrome and all its components. Furthermore, Madeira and cols. consider only absolute serum leptin levels in their paper. It has been observed that leptin levels adjusted to fat mass, but not absolute leptinemia, were correlated to the severity of metabolic syndrome in adults, suggesting a state of relative leptin deficiency in obesity associated with more advanced stages of metabolic syndrome (12).

The authors correctly present two receiver operating characteristic (ROC) curves for the determination of optimal cutoff values, one based on a crude model (i.e., obtained by pooling together all available data), and another based on a sex- and age-adjusted model. When constructing a ROC curve where the accuracy of a diagnostic test (leptin) is affected by covariates (sex and age), failure to incorporate information furnished by them may lead to erroneous conclusions (13). Since it is well-known that leptin levels behave differently according to both age and gender (even in prepubertal individuals) (14), the optimal serum leptin cutoff seems to be indeed 13.4 ng/mL, based on the adjusted model.

However, the construction of a ROC curve provides a fixed cutoff value where sensitivity and specificity are optimal (i.e., the point in the curve closest to the upper left corner, and farthest from the diagonal line). When applying cutoff values to public health, one has to take into account the prevalence of the disorder being tested, and the purpose of the test. For example, a disorder that is very low prevalent and that leads to an unacceptable increase in costs in case of false-positive diagnoses may require the selection of a cutoff that maximizes specificity. On the other hand, if a disorder is highly prevalent, and if missing a diseased individual leads to serious consequences, a lower cutoff value should be selected, to maximize sensitivity. If a test is used for screening purposes, then a higher cutoff value with higher sensitivity and negative predictive values must be used (15). In other words, the optimal cutoff depends on the prevalence of the disease in a target population, and the consequences of false-positive and false-negative results. For the screening of metabolic syndrome in this Brazilian prepubertal population, a serum leptin cutoff of 13.4 ng/mL seems more adequate than 12.3 ng/mL; based on the aforementioned factors, that cutoff could, however, be adjusted to even higher levels.

The study by Madeira and cols. has the merit of studying prepubertal young children, a population that is not widely assessed for metabolic syndrome and the components associated with it. Important descriptive data for that population are presented (which can be used as a reference in future studies), and a cutoff for leptin is proposed for the diagnosis of metabolic syndrome. However, the title may be a bit misleading, since the proposed cutoff does not allow the prediction of the development of metabolic syndrome – the cross-sectional results are merely descriptive, and do not provide long-term information on the development of cardiovascular disease and diabetes – the outcomes that ultimately matter. Only long-term, prospective studies through late adulthood would really answer questions that are relevant to prepubertal children: 1) can their serum leptin levels predict the development of cardiovascular disease and diabetes? 2) if yes, what cutoff should be used, so early intervention can be adopted? At least in adults, the answer to the first question seems to be “probably not” (16,17).

The study by Stroescu and cols. involves cross-sectional data on 122 obese and 42 nonobese Romanian children from 4 to 20 years old, who were further categorized as born small for gestational age (SGA) or appropriate for gestational age (AGA). As previously observed, CIMT was increased among obese children. When analyzing only that group, obese SGA children had higher CIMT values than those born AGA. Subsequently, the authors claimed direct correlation between CIMT and leptin, and between CIMT and high sensitivity C-reactive protein (hsCRP). Inverse weak correlation was described between CIMT and adiponectin. These correlations led the authors to try to determine the optimal CIMT cutoff value that is associated with metabolic syndrome in that population. In that case, a cutoff equal to 0.049 cm is proposed, above which obese children would have increased risk of developing metabolic syndrome.

In this paper, instead of using leptin as a biomarker of metabolic syndrome, the authors chose to employ CIMT – a marker of atherosclerosis that has been associated with the components of metabolic syndrome, and able to predict cardiovascular risk (18). In their statement, the Working Group on Cardiovascular Prevention of the Association for European Pediatric Cardiology “recommends use CIMT in screening patients with elevated cardiovascular risk, even if the long-term benefit of CIMT measurement on the single patient’s vascular health remains to be determined” (19). These observations strengthen the importance of measuring CIMT in obese children.

In the literature, there are heterogeneous results regarding leptin, adiponectin and hsCRP levels in children born SGA (20-22). In the present cohort, SGA obese children had increased CIMT, leptin and hsCRP, and decreased adiponectin, when compared to their obese AGA counterparts. Although the sample size is very small, the results strengthen the hypothesis that SGA babies are at higher risk of developing metabolic syndrome and its consequences (23), mainly due to a low-grade inflammatory state and to altered adipokines levels, independent of adiposity.

It is unclear which criteria the authors used for the diagnosis of metabolic syndrome in the Romanian cohort. As pointed out by Madeira and cols., there is a lack of consensus definition for metabolic syndrome (4). Therefore, not outlining the criteria used to diagnose metabolic syndrome can seriously compromise the validity of the results. Similar to the points made herein to the article by Madeira and cols., the study by Stroescu and cols. is not prospective. Therefore, it cannot propose CIMT as a predictor for future development of metabolic syndrome. Furthermore, the presented coefficients of correlation between CIMT and leptin, adiponectin and hsCRP must be interpreted with caution, since they suggest only weak to moderate correlation. The accuracy of the results presented in Table 2 may be questioned due to the fact that the mean age of the obese AGA group is different from the mean age of the same group that is presented in an article based on data from the same cohort (24). Finally, the authors cannot answer the question posed in the title, due to the small sample size; indeed, they had to combine SGA and AGA data to construct the ROC curve. In their conclusion, the authors do not answer to their own question posed in the title; they expand the most potentially significant result to all obese children, and recommend screening for metabolic syndrome in all of those with CIMT above 0.049 mm, not only SGA. Due to the limitations of the study, it is advisable that this recommendation is not followed until larger, prospective studies are conducted.

The screening of diseases can certainly impose economic burden to individuals and governments. When proposing the use of serum leptin levels or CIMT for the screening of metabolic syndrome, it is necessary to discuss its cost-effectiveness in terms of their ability to predict cardiovascular disease and diabetes in that population. Is it viable to use those tests for the screening of metabolic syndrome in children, considering that the components of metabolic syndrome are invariably measured in the management of obesity (25)? Possibly not. Most importantly, before trying to define a new test for diagnosing metabolic syndrome, researchers should first try to solve the underlying controversy: is metabolic syndrome an entity *per se* carrying a single weight as a risk factor, or is it a constellation of different conditions carrying different risks?
